# The prevention of osteoporotic vertebral fractures in eastern and in western countries

**DOI:** 10.1016/j.bonr.2025.101851

**Published:** 2025-05-15

**Authors:** Fjorda Koromani, Jiawei Li, Hiroshi Hagino, Richard Eastell, Annegreet Vlug, Ling Wang, Hua Yue, Yong-Chan Ha, Steven Cummings, Salvatore Minisola, Claus-C. Glüer, Ling Oei, the East Meets West Action Group of the European Calcified Tissue Society

**Affiliations:** a Department of Internal Medicine, Erasmus MC, Rotterdam, the Netherlands; b Faculty of Medicine, School of Health Science, Tottori University, Japan; c Division of Clinical Medicine, University of Sheffield, Sheffield, UK; d Department of Internal Medicine, Jan van Goyen Medical Center, Amsterdam, the Netherlands; e Department of Internal Medicine, Leiden University Medical Center, Leiden, the Netherlands; f Department of Radiology, Beijing Jishuitan Hospital, Capital Medical University, Beijing, China; g Department of Osteoporosis and Bone Diseases, Shanghai Jiao Tong University Affiliated Sixth People's Hospital, Shanghai, China; h Department of Orthopaedic Surgery, Seoul Bumin, Hospital, Seoul, South Korea; i San Francisco Coordinating Center, California Pacific Medical Center Research Institute, San Francisco, CA, USA; j Departments of Epidemiology and Biostatistics, University of California, San Francisco, CA, USA; k Department of Radiology, Peking University Jishuitan Hospital, Beijing, China.; l Department of Clinical, Internal, Anesthesiologic and Cardiovascular Sciences, Sapienza University of Rome, Rome, Italy; m Section Biomedical Imaging, Department of Radiology and Neurology, University Hospital Schleswig-Holstein, Kiel, Germany; n Department of Epidemiology, Erasmus MC, Rotterdam, the Netherlands

## Abstract

Osteoporotic vertebral fractures (VFs) are among the most common and clinically significant manifestations of skeletal fragility, contributing substantially to morbidity, disability, and future fracture risk worldwide. Yet, their recognition and management remain inconsistent across regions. To explore differences and similarities in the prevalence, diagnosis, management, and prevention of vertebral fractures, the East Meets West (EmW) Action Group of the European Calcified Tissue Society convened a multi-country exchange among clinical and research experts from Europe, the USA, and East Asia. This report summarizes the discussions and synthesizes current knowledge on the topic. Evidence from China, South Korea, Japan, and Germany shows a wide range in reported VF prevalence and incidence, largely influenced by differences in population aging, imaging access, and diagnostic adjudication methods. While lateral spine radiographs remain the standard for detection in both research and clinical care, variable use of quantitative morphometry (QM), semi-quantitative (SQ), and algorithm-based qualitative (ABQ) methods limits comparability. MRI remains the gold standard for assessing fracture acuity, but is not feasible for widespread screening. VFA via DXA is gaining popularity, although underutilized in several settings. Despite the availability of effective pharmacologic treatments, including bisphosphonates, denosumab, and anabolic agents, treatment rates following VF remain suboptimal across all countries studied. None of the countries currently has a nationwide vertebral fracture screening program, although fracture liaison services (FLS) and AI-assisted imaging offer promising pathways forward. The lack of a universally accepted definition and gold standard for VF adjudication continues to hamper clinical decision-making and data harmonization. This report highlights the need for greater international consensus on diagnostic criteria, improved integration of vertebral fracture screening into clinical workflows, and the development of targeted strategies to close treatment gaps and reduce the global burden of vertebral fractures.

## 1 Introduction

Osteoporosis, characterized by low bone mass and micro-architectural deterioration, is a global health concern, affecting approximately 200 million people worldwide and results in 8.9 million osteoporosis-related fractures annually (Barnsley et al., 2021; *Am. J. Med.*, 1991). Osteoporotic vertebral fractures are prevalent among the middle-aged and elderly population. They pose a global health issue due to their association with increased morbidity, subsequent vertebral and non-vertebral fractures, and mortality. Therefore, the East Meets West (EmW) Action Group of the European Calcified Tissue Society (ECTS) has met on this topic and has organized an exchange of experiences and insights between clinicians and researchers from several countries from USA, Europe and East-Asia. Specifically, following an online ECTS educational activity and panel discussion in the year 2021, participants from Asia, the US, and Europe agreed on key points to recognize that are presented in this contribution. Following this event, the writing of a collaborative meeting report had become a recurring topic in the annual East meets West Action Group's committee in-person meetings These key points include: the clinical relevance of osteoporotic vertebral fractures in general; their specific radiological definition and diagnosis; prevalence and adverse health effects of vertebral fractures observed in different countries such as China, Japan, Korea and Germany; and how different national guidelines may advise in management and ideally prevention of these osteoporotic vertebral fractures.

### 1.1 Impact of prevalent vertebral fractures on subsequent factures

Vertebral fractures are common, and a higher incidence compared to other fracture locations is observed in both men and women (Sambrook and Cooper, 2006). Both clinical and radiographic vertebral fractures are associated with an increased risk of disability and future clinical vertebral and non-vertebral fractures. In the Study of Osteoporotic Fractures, 7223 women were followed up for an average of 3.7 years, and it was observed that those who sustained an incident vertebral fracture during follow-up had an increased relative risk of back pain, limited activity and disability (Nevitt et al., 1998). Similarly, in the study by Fink et al. women with a clinical vertebral fracture had a similar time of disability to that following a hip fracture (Fink et al., 2003). Treatments have different efficacy for vertebral fracture prevention; in the network meta-analysis by Barrionuevo et al. including 107 trials (193,987 postmenopausal women; mean age, 66 years; of which 55 % white and the remaining 45 % of several ancestral backgrounds including Asian, African-American and Hispanic). It was reported that zoledronate, denosumab, romosozumab, teriparatide and abaloparatide had higher efficacy compared to alendronate, risedronate or raloxifene (Barrionuevo et al., 2019). Identifying individuals at increased risk of vertebral fracture could involve risk factor identification or imaging. The presence of vertebral fracture on imaging indicates a 3–5-fold increase in the risk of subsequent vertebral fracture and other fractures, depending on number and grade of the prevalent vertebral fractures (Black et al., 1999). This supports the argument that identifying individuals at high risk of vertebral fracture via screening by imaging could be a good and effective approach. Questions to address: a) how common are vertebral fractures b) by what method are they ascertained? c) If common, do guidelines include screening by vertebral imaging? d) when a vertebral fracture is identified, do guidelines emphasize the most effective treatments for preventing vertebral fracture? e) should guidelines be revised to include specific screening and treatment for vertebral fractures?

### 1.2 Image-aided diagnosis of vertebral fractures

While MRI is the gold standard for assessing the temporality of vertebral fracture, particularly in distinguishing acute from chronic fractures based on bone marrow signal change, it is not routinely used for screening or research purposes. For the identification of asymptomatic vertebral fractures or for use in large-scale epidemiological studies, lateral spine radiographs remain the preferred method due to their accessibility, standardization, and cost-effectiveness. Vertebral fracture assessment (VFA) on lateral Dual X-ray absorptiometry (DXA) has also gained ground as low-radiation, efficient alternative, particularly in clinical practice. However, it remains less accurate than conventional radiography, especially for upper thoracic vertebrae or in patients with high body mass index. Importantly, both radiographic and VFA-based approaches are limited by the absence of a universally accepted gold standard for vertebral fracture adjudication, which contributes to variability in reported prevalence and fracture classification (Oei et al., 2013a). Long before the advent of systematic approaches, vertebral fracture diagnosis was inconsistent across different readers. To introduce improved consistency, approaches that are more systematic were introduced, such as quantitative morphometry (QM), semi-quantitative (SQ) and algorithm based qualitative (ABQ) methods, which we will discuss here. In QM, the dimensions of the vertebra are measured directly (anterior, middle and posterior heights) and used to calculate height ratios. A vertebra is classified as fractured if there is a height reduction compared to the adjacent vertebrae or compared to expected norms (Black et al., 1999; Eastell et al., 1991; McCloskey et al., 1993). Then fractures are graded further by percentage loss to grade 1 (20–25 %), grade 2 (25–40 %) and grade 3 (>40 %). QM is reproducible, widely used in clinical trials and large datasets, and objective, allowing for shape and size variability. Drawbacks are, however, that it may be more laborious and time-consuming, that there is a high chance of false positives especially for grade 1, and no differential diagnosis of deformities is given. Nevertheless, distinction of these false positives is important, because this can have therapeutic consequences. From previous studies in Europe it is known that prevalences of non-fracture deformities may vary geographically (Armbrecht et al., 2015). SQ on the other hand, adds some qualitative assessment in the reading by introducing characterization of shape, endplate deformity and cortical disruption to the QM method (Genant et al., 1993). However since the method does not rely on direct measurements, it can be reader dependent, requires more training and is less suitable for automation (Genant et al., 1993).

**Fig. 1:**
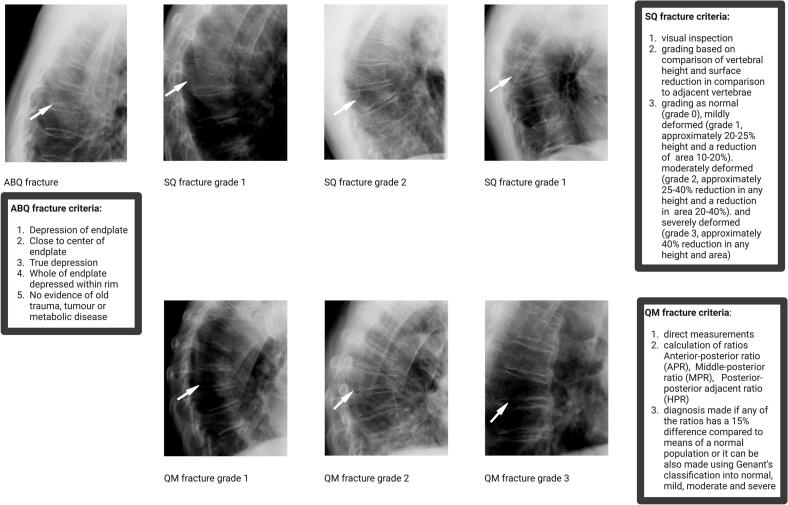
Overview of vertebral fracture assessment criteria: ABQ (Algorithm-Based Qualitative), SQ (Semiquantitative), and QM (Quantitative Morphometry) methods.

**Fig. 2:**
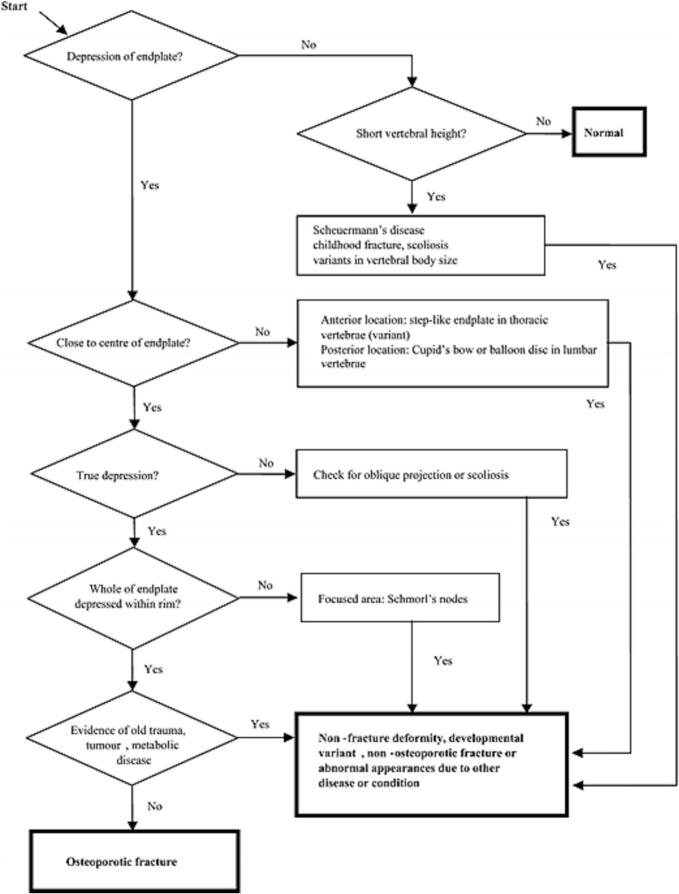
Diagnostic flowchart for osteoporotic vertebral fracture.

A drawback of both QM and SQ is that they lack a clear description of a differential diagnosis with vertebral deformities There is no agreement whether short anterior height vertebrae with intact endplates are fractures. Evidence that short vertebral height (SVH) is just as common in younger (20–40 years) as in older (55–88 years) women and lack of association with subsequent vertebral fractures, points toward SVH not being true fractures (Ferrar et al., 2012). The ABQ method is a visual tool requiring endplate involvement to call a fracture, has the advantage of no minimum threshold for the apparent reduction in vertebral height. Basically a vertebral fracture is called only if there is endplate depression, or vertebral wall buckling or evidence of fracture lines (Jiang et al., 2004). The decision tree helps to make the method systematic and reproducible. While both QM and SQ methods are commonly used to detect vertebral fractures in clinical and research settings, they differ in sensitivity and specificity. QM relies solely on vertebral height measurements and may misclassify normal variants as fractures. SQ adds expert visual judgment, incorporating shape and cortical changes to improve accuracy. The ABQ method further refines this by requiring radiographic evidence of endplate or cortical disruption, thereby reducing false positives. Though more specific, ABQ may miss milder fractures and requires experienced readers, limiting its widespread adoption in large-scale studies. As expected prevalence and incidence of vertebral fractures diagnosed with SQ were higher than those diagnosed with ABQ (Oei et al., 2018). Furthermore, ABQ-diagnosed vertebral fractures are associated with higher risk of both vertebral and non-vertebral fractures compared to SQ-diagnosed vertebral fractures (Koromani et al., 2020; Lentle et al., 2018). What exactly is an osteoporotic vertebral fracture? Fairly comprehensive collection of image examples have been published in pictorial reviews by Wáng et al (Wáng et al., 2018; Wáng et al., 2017). The anatomy of the vertebral body also has been investigated using data from clinical trials. When looking back at clinical trial images, it was noticed that in all incident vertebral fracture cases, there was a deformity of the endplate involved. To localize the endplate in a normal vertebra, we need to identify the vertebral ring line and the central endplate overlapping with the ring endplate. In osteoporotic vertebral fractures, there is evidence of depression of the central endplate with or without a fracture of the vertebral ring or fracture of the cortex of the vertebral body (Jiang et al., 2004). > 90 % of the vertebral fractures occur in the superior endplate.^20^(Fig. 1) Besides diagnosing on lateral radiographs and DXA, vertebral fractures can be assessed on computed tomography (CT) scan, including those performed for indications other than osteoporosis (e.g. chest and abdomen CT for cancer diagnosis). Because of the very large number of CT examinations performed worldwide, automated “opportunistic” assessment of such CT data could substantially improve detection rates of vertebral fractures. However, standardization of CT-based assessment of vertebral fractures has not been undertaken. For any imaging modality the evaluation of vertebral fracture could be improved by using computer-supported approaches (Oei et al., 2013b). Recent academic efforts have increasingly focused on developing artificial intelligence (AI) tools to automate the detection of vertebral fractures on lateral spine radiographs and DXA-derived images. Several research groups have trained deep learning models—most commonly convolutional neural networks (CNNs) on large annotated datasets using Genant's semi-quantitative method as a reference standard. (Dong et al., 2022; Seo et al., 2021; Kim et al., 2021) These models aim to replicate or even surpass expert-level performance in identifying moderate-to-severe vertebral fractures. For example, a study by Murata et al. demonstrated that a deep CNN achieved accuracy, sensitivity, and specificity rates of 86.0 %, 84.7 %, and 87.3 %, respectively, for detecting osteoporotic vertebral compression fractures on lateral spine radiographs (Murata et al., 2020). In the DXA domain, academic collaborations have explored AI-enhanced analysis of VFA images to reduce false positives and improve throughput. One study showed that AI-derived vertebral fracture classifications on VFA predicted future clinical fractures with similar validity as those made by expert readers (Monchka et al., 2021). However, a key limitation in both training and validating these models lies in the absence of a universally accepted gold standard for vertebral fracture adjudication. This raises a fundamental concern related to the principle of “garbage in, garbage out” (GIGO): AI models are only as reliable as the quality and consistency of the input data used for training. Inconsistent or subjective labeling, particularly in mild deformities or cases lacking clear cortical disruption, can propagate errors and reduce generalizability across settings and populations. Efforts to automate the ABQ method, which requires visual confirmation of cortical or endplate disruption, are still in early phases. Due to the nuanced visual criteria and need for high-resolution imaging, ABQ is more difficult to replicate algorithmically compared to height-based or semi-quantitative methods. To date, there are no fully validated AI tools that automate ABQ classification at scale, though research in this direction is ongoing (Li et al., 2024). (See Fig. 2.) (See Table 1.)

**Table 1:** Summary of studies assessing vertebral fractures in Eastern and Western countries.

Country	Publication	Vertebral fracture assessment method	Sample size	Sex and age range	Prevalence / incidence (95 % confidence interval)
China	Ling et al., 2000	QM	102	Women 50–59 years	*P* = 4.9 % (0.7 %–9.1 %)
			105	Women 60–69 years	*P* = 16.2 % (9.1–23.2 %)
			100	Women 70–79 years	*P* = 19.0 % (11.3–26.7 %)
			93	Women >80 years	*P* = 36.6 % (26.8 %–46.3 %)
China	Cui et al., 2017	SQ	1760	Women <60 years	*P* = 13.4 %
				Women 60–69 years	*P* = 22.6 %
				Women 70–79 years	*P* = 31.4 %
				Women >89 years	*P* = 58.1 %
China	Kwok et al., 2013	SQ	2000	Women (mean age 72.6 years)	*P* = 16.5 %
			2000	Men (mean age 72.4 years)	*P* = 14.9 %
China	Wang et al., 2020	Self-reported	3457	Women and men (62.7 % women)	P = 16.5 %
China	Xia et al., 2022	SQ	2634	Women 50–54 years	*P* = 0.9 % (0.0 %–2.2 %)
				Women 55–59 years	*P* = 4.2 % (2.2 %–6.1 %)
				Women 60–64 years	*P* = 9.4 % (6.9 %–11.9 %)
				Women 65–69 years	*P* = 13.3 % (10.2 %–16.4 %)
				Women 70–74 years	*P* = 20.9 % (17.0 %–24.8 %)
				Women 75–79 years	*P* = 23.7 % (19.3 %–28.2 %)
				Women >80 years	*P* = 35.7 % (29.6 %–41.7 %)
China	Wang et al., 2021	SQ	4834	Women 40–49 years	*P* = 1.5 % (0.8 %–2.3 %)
				Women 50–59 years	*P* = 6.2 % (4.5 %–8.0 %)
				Women 60–69 years	*P* = 15.5 % (12.7 %–18.2 %)
				Women 70–79 years	*P* = 28.1 % (21.7 %–34.5 %)
				Women >80 years	*P* = 38.1 % (22.1 %–54.1 %)
			3589	Men 40–49 years	*P* = 3.2 % (1.0 %–5.4 %)
				Men 50–59 years	*P* = 11.1 % (8.5 %–13.7 %)
				Men 60–69 years	*P* = 14.3 % (11.6 %–16.9 %)
				Men 70–79 years	*P* = 23.8 % (18.7 %–29.0 %)
				Men >80 years	*P* = 36.0 % (20.1 %–51.8 %)
China	Zhao et al., 2024	SQ	10,095	Women ≥ 40 years	*P* = 10.7 %
					P = 4.2 % (≥ grade 2)
			7394	Men ≥ 40 years	*P* = 8.7 %
					*P* = 3.6 % (≥ grade 2)
Korea	Shin et al., 2012	QM	298	Women 40–49 years	*P* = 4.4 %
			449	Women 50–59 years	P = 8.7 %
			565	Women 60–69 years	P = 20.9 %
			217	Women 70–79 years	*P* = 26.3 %
			200	Men 40–49 years	*P* = 6.6 %
			391	Men 50–59 years	*P* = 7.9 %
			389	Men 60–69 years	*P* = 14.8 %
			169	Men 70–79 years	*P* = 20.1 %
Korea	Park et al., 2016	Medical record coding	72,857	Women	*I* = 656/100.000 person years
				Men	*I* = 248/100.000 person years
Japan	Tsukutani et al., 2015	Clinical vertebral fractures on X-ray	36,167	Women >50 years	*I* = 1229/100.000 person years
				Men >50 years	*I* = 412/100.000 person years
Japan	Hamasaki et al., 2020	Insurance database coding	234,613	Women >65 years	*I* = 21.2/1000
				Men >65 years	*I* = 7.29 / 1000
Japan	Fujiwara et al., 2003	SQ	2356	Women 47–95 years	*P* = 9.5 %
				Women <49 years	*I* = 3.2/1000 person years in absence of prevalent vertebral fracture; 27.4/1000 person years in presence of prevalent vertebral fracture
				Women 50–59 years	*I* = 5.2/1000 person years in absence of prevalent vertebral fracture; 36.2/1000 person years in presence of prevalent vertebral fracture
				Women 60–69 years	*I* = 12.4/1000 person years in absence of prevalent vertebral fracture; 59.7/1000 person years in presence of prevalent vertebral fracture
				Women 70–79 years	*I* = 24.5/1000 person years in absence of prevalent vertebral fracture; 88.0/1000 person years in presence of prevalent vertebral fracture
				Women >80 years	*I* = 56.1/1000 person years in absence of prevalent vertebral fracture; 141.0/1000 person years in presence of prevalent vertebral fracture
				Men 47–95 years	P = 3.2 %
				Men <59 years	*I* = 2.5/1000 person years in absence of prevalent vertebral fracture; 19.4/1000 person years in presence of prevalent vertebral fracture
				Men 60–69 years	*I* = 6.5/1000 person years in absence of prevalent vertebral fracture; 31.4/1000 person years in presence of prevalent vertebral fracture
				Men 70–79 years	*I* = 12.8/1000 person years in absence of prevalent vertebral fracture; 44.8/1000 person years in presence of prevalent vertebral fracture
				Women >80 years	*I* = 25.9/1000 person years in absence of prevalent vertebral fracture; 64.2/1000 person years in presence of prevalent vertebral fracture
Germany, UK and France	Ferrar et al., 2012	ABQ	674	Women 55–80 years	P = 7 %; I = 3 % for 6 years of follow-up
USA	Ferrar et al., 2007	ABQ	9516	Men >65 years	P = 10 %
		SQ	9516	Men >65 years	P = 13 %

### 1.3 Vertebral fractures in China

In China, the growing burden of vertebral fractures reflects the country's rapidly aging population and evolving healthcare landscape. The prevalence of VFs in participants of the cohort studies Mr.Os Hong Kong and Ms. Os Hong Kong, was reported as 14.9 % among men and 16.5 % among women aged on average 72.4 years old and defined based in Genant's SQ adjudication method. Of note: majority of VF among men were grade 1. (Kwok et al., 2013) In a random sample of postmenopausal women from Bejing, SQ adjudicated VF had a prevalence of 13 % in women aged between 50 and 59 years old, whereas the prevalence increased to 50 % in women aged >80 years old (Cui et al., 2017). In a randomly selected sample (*n* = 2634) across 10 Tier-3 hospitals spanning 5 regions, prevalence of VF adjudicated by the SQ method was 14.5 % (Xia et al., 2022). In a thorough analysis among adults aged 40 years and older, the prevalence of VF was reported 10.5 % in men and 9.7 % in women, as determined by radiographic assessments using Genant's semi-quantitative method, thus showing no sex dimorphism in VF prevalence (Wang et al., 2021). As expected the prevalence increased with age for both sexes, surpassing 35 % in individuals aged older than 80 years old. However the data showed a higher prevalence of VF in men residing in rural areas (11.8 %) compared to those residing in urban areas (8.2 %). There have been attempts to compare prevalence of VF in China vs. western populations by standardizing the adjudication method employed and matching the population characteristics. For example, Ling et al. performed vertebral fracture readings according to both Eastell et al. and Black et al. adjudication methods in a random sample of 402 Chinese women aged 50 years and above in Beijing and compared prevalence with that of the Study of Osteoporotic Fractures (SOF) and women in Rochester, MN, U.S.A (Ling et al., 2000). The prevalence of vertebral fractures defined in lateral radiographs with QM by the methods of Eastell et al. was 4.9 % in Chinese women between 50 and 59 years old and 36.6 % among women above 80 years old. Overall, and also when stratified by age, prevalence of VF was slightly lower in Chinese women compared to women from the SOF and Rochester studies. Wang et al. also attempted to directly compare prevalence of VF in Chinese women vs. Women residing in Rome, Italy, by employing similar sampling and adjudication methods for VF. Among age-matched (mean: 74.1 years) women, they found a prevalence of 38.5 % in Chinese women compared to 61.5 % in Italian women (Wáng et al., 2021). With regards to the incidence of VFs, there is evidence that in urban China it has risen from 85.21 per 100,000 person-years in 2013 to 152.13 per 100,000 person-years in 2017, with women exhibiting nearly three times the incidence rate of men (Zheng et al., 2023). Management of VFs in China is guided by national consensus guidelines recommending pharmacologic treatment, including antiresorptive or anabolic agents, for patients with diagnosed osteoporotic VFs. However, treatment rates remain low, with studies indicating that only 0.3 % of men and 1.4 % of women with osteoporosis or fractures receive anti-osteoporosis treatment. Barriers to effective management include underdiagnosis, limited access to healthcare resources, and low public awareness (Cui et al., 2017; Yu and Xia, 2019). Currently, China lacks a nationwide screening program for vertebral fractures, though there is growing interest in integrating VF assessments into fracture liaison services (FLS) and utilizing artificial intelligence (AI) tools to aid detection in clinical settings. The absence of standardized adjudication methods and screening protocols hampers the development of cohesive strategies for VF prevention and management.

### 1.4 Vertebral fractures in South Korea

South Korea has seen a sharp rise in vertebral fracture cases over recent decades, underscoring the country's demographic transition and increased awareness of osteoporosis. A community-based study reported a standardized prevalence of VFs at 8.8 % in men and 12.6 % in women aged 43–74 years, assessed using vertebral morphometry on lateral spine radiographs (Kim et al., 2016). In the Ansyung cohort, a community-based prospective study among 2684 participants, vertebral fractures were scored from T11-L4 on lateral radiographs (Jang et al., 2016). The prevalence of vertebral fractures among men was 11.9 % and 14.8 % among women. National data from 2008 to 2012 indicated that the incidence of spinal fractures among individuals aged 50 years and older increased from 603 to 953.1 per 100,000 person-years in women and from 245.3 to 312.5 per 100,000 person-years in men. The incidence was considerably higher in women, with rates approximately three times greater than in men (Kim et al., 2016; Shin et al., 2012). In the overview by Ballane et al. women in South Korea had the highest age-standardized incidence of vertebral fractures ranked against other countries (Shin et al., 2012; Ballane et al., 2017). The number of vertebral fractures is expected to increase in 2025 by 60 % in men and 50 % in women, respectively (Kim et al., 2016). Management strategies for VFs in South Korea are guided by fracture risk stratification. The Korean Society for Bone and Mineral Research recommends categorizing patients into low, moderate, high, and very high-risk groups based on factors such as recent fractures, bone mineral density (BMD), and FRAX scores among others (Park et al., 2023). Treatment options include antiresorptive agents like bisphosphonates and denosumab, as well as anabolic agents for those at very high risk. Despite these guidelines, treatment rates remain suboptimal, with only a fraction of patients receiving appropriate osteoporosis therapy post-fracture. (Ahn et al., 2020) Screening programs for osteoporosis and VFs are limited but evolving. The National Health Insurance Service provides BMD screening for women aged 66 years as part of the National Screening Program for Transitional Ages (Kim et al., 2012). However, systematic VF screening is not yet widespread. It is important to note some aspects of the Korean National Health Insurance (KNHI) system; 100 % of the population is insured and KNHI covers 70 % of total costs and the remaining 30 % comprises exceptions such as cosmetic surgeries or accidents covered by other insurers. In South Korea, insurance covers osteoporosis medication if the DXA-BMD T-score at the hip or spine is ≤ −2.5. If the patient suffers an osteoporotic fracture, there is reimbursement for anti-osteoporosis treatment for three years. This is irrespective of DXA, and rh-PTH is classified as an additive option to treat osteoporosis. There has been a proposal in a public hearing with the national assembly to extend insurance reimbursement. Two proposed changes: 1. Maintaining period after improving from osteoporosis to osteopenia and 2. Introducing a new reimbursement system where osteopenia and fracture risk assessment (FRAX) are considered. To prevent subsequent vertebral fractures, effort has been placed in fracture liaison services (FLS). A blue book has been written and distributed and a training and education for coordinators of FLS has been set up (Lim et al., 2023).

### 1.5 Vertebral fractures in Japan

Japan, with one of the oldest populations globally, has long recognized vertebral fractures as a critical component of its national osteoporosis burden. Studies using Genant's SQ method on lateral spine radiographs have reported VF prevalence rates of approximately 17.8 % among elderly Japanese residing in rural areas (Yamada et al., 2023). Importantly, the prevalence of VFs has decreased over the past two decades, likely due to improvements in bone mineral density and osteoporosis treatment rates. (Yamada et al., 2023) Age-specific prevalence of vertebral fractures in Japanese women is found to be slightly higher than in China mainland and Taiwan, Hawaii and Minnesota in the USA (Hagino, 2021). The differences can be partly explained by the different definitions used to define vertebral fractures. The incidence of clinical VFs increases with age and is higher in women. For instance, in Kure City, the incidence rate per 1000 person-years among individuals aged 65 and older was 15.58 overall, with 7.29 in men and 21.17 in women (Hamasaki et al., 2020). These figures underscore the gender disparity and the heightened risk among older adults. Among 36,167 inhabitants of Sakaiminato City in Japan, the incidence of clinical vertebral fractures was reported 27 % during a follow-up of 2 years (Tsukutani et al., 2015). The incidence rate among men reached 2000/100,000 person years and 3500/100,000 person years in women of 80 years old. In an overview made to compare incidence rates between eastern populations (Hong Kong, South Korea, Japan) and western populations (Sweden, USA) the incidence rates in Eastern populations are higher compared with the Western populations. Management strategies for VFs in Japan are guided by national guidelines. (Hamasaki et al., 2020; Bow et al., 2012; Lee et al., 2012; Kanis et al., 2000; Cooper et al., 1992) The 2011 Japanese guidelines for the prevention and treatment of osteoporosis recommend pharmacologic treatment, including antiresorptive or anabolic agents, for patients with diagnosed osteoporotic VFs (Orimo et al., 2012). However, treatment rates remain suboptimal. A study found that only 43.6 % of patients with vertebral fractures received bone mineral density testing after the fracture, and 61.7 % received osteoporosis treatment within 12 months (Nakatoh et al., 2021). In Japan, the diagnostic work-up for osteoporosis starts with an assessment of the fragility fracture status. Vertebral fractures are first defined on lateral radiographic images of the spine. In the presence of clinical complaints such as back pain, and inconclusive radiographic images, a CT scan is performed and is then used to define vertebral fractures. If the CT is also inconclusive, vertebral fractures might be defined through MRI examination. In the presence of prevalent hip or vertebral fractures a working diagnosis of primary osteoporosis is established. If other types of fractures are identified, then BMD is assessed and osteoporosis is diagnosed if BMD is at least 20 % lower than that of a young adult man. In the absence of fragility fractures, osteoporosis is diagnosed if BMD is 30 % or more decreased compared to a young adult man or if the T-score is below −2.5 (Soen et al., 2013). Screening programs for osteoporosis and VFs are evolving. Japan has implemented the Osteoporosis Liaison Service (OLS) since 2011, focusing on primary and secondary prevention of fractures. Additionally, the Fracture Liaison Service (FLS) reimbursement scheme was updated in 2022 to improve post-fracture care and decrease the burden of fragility fractures (Gondo et al., 2025). Nevertheless, systematic VF screening is not yet widespread, and underdiagnosis remains a challenge.

### 1.6 Vertebral fractures in Germany

In Germany, vertebral fractures have gained increasing attention as hospitalization rates rise and clinical guidelines prioritize early identification and treatment. Between 2009 and 2019, the incidence of hospitalized VFs rose by 45.6 %, reaching 150.7 per 100,000 inhabitants (Lang et al., 2023). The lumbar spine was most commonly affected, accounting for 46.8 % of cases. Notably, 63.7 % of VFs occurred in women, and 69.0 % were diagnosed in individuals aged 70 years or older. In the German centers of the Osteoporosis and Ultrasound Study (OPUS), the prevalence of radiologic vertebral fractures defined as height reduction above 20 % ranged from 8.1 % in the 50–59 years group to 27 % in the 75–79 years group. However, the incidence of vertebral fractures is more difficult to estimate, and several approaches have been proposed, such as the ratio of vertebral/hip fracture incidence, which was used in earlier version of the guidelines to estimate vertebral incidence rate from well-established hip fracture incidence data, inception cohort studies, register data, insurance register data and local surveys (Lam et al., 2014). When examining the incidence of vertebral fractures in the OPUS study an incidence of radiographic vertebral fractures of 3 % for 6 years of follow-up is reported in postmenopausal women (Ferrar et al., 2012). The radiographic vertebral fracture incidence estimation was recently refined using the insurance registers representative of the German population. 3-year incidence rates in women increase from about 1 % at 60 to >4 % at age 80, with men experiencing incidence rates at about half this level. The management of vertebral fractures is based on the German Osteoporosis Guidelines as developed by the Dachverband Osteologie (DVO) a federation of about twenty societies working in different aspects of bone health in German-speaking countries (Germany, Austria, and German-speaking Cantons of Switzerland). Because of the relevance of vertebral fracture, in these guidelines therapy decisions have long been based on risk of osteoporotic fractures of the spine or hip, rather than major osteoporotic fractures (MOF) or hip fractures alone. Aside of osteoporosis guidelines the DVO has set up certification programs for physicians and surgeons to become a certified “Osteologe” (osteologist, i.e. expert on bone) and for expert osteology centers. From the ECTS EmW symposium 2020 a comparative review was made of osteoporosis case ascertainment strategies in European and Asian countries (Burden et al., 2021; Pfeil and Lange, 2024). These procedures have been revised in the 2023 guidelines just released (Pfeil and Lange, 2024). To decide on therapy commencement in Germany, patients have to have one of 33 clinical risk factors identified in the guideline. 3-year fracture risk of hip or vertebral fracture is calculated and therapy is indicated based on three specific thresholds: therapy proposed (3 %), therapy advised (5 %), bone-anabolic therapy advised (10 %). Independent of these risk thresholds, therapy is also indicated if a hip fracture is identified. Hip fractures may, however, often occur quite late in life and therefore, more criteria have been selected. If vertebral fractures are suspected, spinal radiography or VFA is performed, and if a vertebral fracture is identified (Genant grade 2 or higher, or two Genant grade 1), then therapy is indicated. If a patient has been using glucocorticoids 7.5 mg/day or more for 3 months or longer, then therapy is also indicated. Screening programs for osteoporosis and VFs are limited but evolving. Currently, Germany lacks a nationwide screening program for vertebral fractures. However, initiatives like the Capture the Fracture® program aim to improve post-fracture care and decrease the burden of fragility fractures.

## 2 Discussion

Vertebral fractures (VFs) are a common and clinically significant consequence of osteoporosis, associated with increased morbidity, disability, risk of subsequent fractures, and mortality. Despite their impact, the recognition, diagnosis, and management of VFs remain inconsistent across countries. This international comparative review by the ECTS East Meets West (EmW) Action Group highlights both the diversity and convergence in approaches across China, South Korea, Japan, Germany, and beyond, emphasizing the urgent need for unified strategies in diagnosis, management, and prevention.

### 2.1 Shared challenges across regions

Across all countries included in this report, several common themes emerge. First, VFs remain underdiagnosed, especially asymptomatic ones, which limits timely intervention. This under recognition is partially attributable to the lack of standardized imaging and adjudication protocols. Radiographic detection remains the cornerstone of VF identification in clinical practice and epidemiological studies, but variable use of semi-quantitative (SQ), quantitative morphometry (QM), and algorithm-based qualitative (ABQ) methods leads to differences in reported prevalence. The absence of a universally accepted gold standard for fracture adjudication not only hampers comparability but also complicates the training of both radiologists and AI algorithms. This reflects the broader challenge of heterogeneity in clinical criteria and thresholds for diagnosis and treatment initiation. Secondly, while MRI is acknowledged as the gold standard for determining the temporality of fractures, distinguishing recent from older events, it is not practical for population-level screening or routine osteoporosis assessment. VFA by DXA, although cost-effective and widely available in some countries, has variable accuracy and limited adoption in others, especially rural areas. Opportunistic VF detection through CT scans taken for unrelated indications (e.g. oncology staging) represents a promising future direction, particularly when combined with AI-based image analysis. Third, while all countries reviewed have established osteoporosis treatment guidelines that include VFs as a high-risk condition, real-world treatment rates remain disappointingly low. The treatment gap is particularly pronounced in China, where <2 % of those with diagnosed osteoporosis or fractures are on active pharmacologic therapy. Even in countries with relatively strong healthcare systems and reimbursement structures, such as Japan and Germany, fracture liaison services (FLS) and guideline-concordant care are not yet universally implemented.

### 2.2 Differences in prevalence and incidence

Country-specific data show marked variation in the prevalence and incidence of vertebral fracture, differences that reflect not only underlying demographics but also adjudication and imaging practices. For example: In *China*, VF prevalence in older adults ranges from ∼10 % to over 50 % depending on age, region (urban vs. rural), and adjudication method. Prevalence is higher in rural men compared to their urban counterparts, suggesting possible disparities in occupational risk or healthcare access. In *South Korea*, vertebral fracture prevalence is consistently higher in women than men, with national data showing incidence rates of over 950 per 100,000 person-years in older women, among the highest globally. This is consistent with high age-standardized incidence rates reported in international comparative reviews. *Japan* shows both high incidence and a decreasing trend in prevalence over time, possibly reflecting improvements in osteoporosis awareness and treatment. However, studies report a wide range of VF incidence figures (from 15 to 3500 per 100,000 person-years), depending on study setting and fracture definition. In *Germany*, the incidence of hospitalized vertebral fractures increased by over 45 % between 2009 and 2019, underscoring the growing burden of fragility fractures in aging populations. However, incidence estimates vary depending on data source, ranging from insurance claims to inception cohort studies and assumptions used to convert hip fracture data into VF estimates. These variations suggest that some reported differences in VF burden may reflect methodological discrepancies more than true epidemiological divergence. Standardization of definitions and adjudication methods is crucial for improving global comparability.

### 2.3 Imaging and adjudication: Need for consensus

The lack of a universally accepted gold standard for vertebral fracture adjudication is a major barrier to clinical and research progress. QM, SQ, and ABQ each have strengths and limitations. QM is objective but prone to false positives, especially for mild deformities. SQ adds visual judgment but is reader-dependent. ABQ is more specific but may miss mild fractures and is harder to automate. The introduction of high-quality pictorial reviews and structured decision algorithms (e.g., those by Wang et al.) has improved educational consistency. However, differences in whether a short anterior vertebral height constitutes a fracture remain unresolved. Notably, studies have shown that “short vertebral height” (SVH) is equally common in younger and older women and not predictive of future fractures, arguing against its classification as a true osteoporotic fracture. AI-based automation of VF detection represents a promising solution, particularly in overburdened healthcare systems. However, these tools depend heavily on the quality of training data. Without standardization of adjudication criteria, AI systems risk perpetuating inconsistent diagnoses, a classic case of “garbage in, garbage out.” Research efforts to train AI using ABQ-like definitions may offer a path forward, though these are still in early stages.

### 2.4 Management gap

Despite clear evidence that vertebral fractures strongly predict future fractures and disability, treatment initiation remains inconsistent. In China, only 0.3 % of men and 1.4 % of women with diagnosed osteoporosis or VFs receive pharmacologic therapy. Similar gaps are observed in South Korea and Japan, though both countries have national risk stratification frameworks that support targeted intervention. Germany stands out for its comprehensive DVO guideline structure, which includes explicit thresholds for initiating therapy based on 3-year risk of hip or vertebral fractures, and recognizes the importance of early identification of Genant grade 2+ VFs. However, even in Germany, osteoporosis care is fragmented, and there is no national VF screening program. Some initiatives, such as certification of osteologists and development of fracture liaison services, may help close the gap, but broader implementation is needed.

### 2.5 Toward unified prevention strategies

A key takeaway from this international comparison is the lack of standardized screening programs for vertebral fractures. Only in South Korea is bone mineral density screening systematically offered to women aged 66 through the national health insurance program. Japan and Germany have made strides toward structured FLS models, while China is exploring the integration of AI tools and tiered hospital systems to enhance detection and follow-up. Importantly, all countries reviewed include VFs in their clinical treatment thresholds, yet none has a population-wide VF imaging screening program. Considering the high predictive value of prevalent VFs for future fractures and the relatively low cost of lateral radiographs or VFA, this represents a missed opportunity for proactive intervention.

## 3 Discussion points raised and potential topics for future research and international EmW collaborations

Our investigation of the importance of vertebral fracture assessments in different countries in East and West revealed similarities and differences. In order to move forward the following list of discussion points were considered of particular relevance. We have provided practical next step proposal for each of the discussion points:

I. The radiological diagnosis of vertebral fractures in osteoporosis: comparison of ABQ, SQ and QM methods. Are population-specific references needed and will further implementation of AI-assisted diagnosis improve global applicability? A potential project to address this point could be the initiation of a multi-country image-sharing project to create a curated, annotated image bank containing vertebral fractures adjudicated by QM, ABQ and SQ methods. This dataset can be used to train and validate AI models and to harmonize interpretations across diverse populations.
II. Could more countries implement DXA-VFA vertebral fracture diagnosis as a first line instead of plain radiographs? A potential project to tackle this could be piloting a DXA VFA first workflow in one or two hospitals per country targeting high-risk patients referred for BMD testing. Collecting data on fracture detection rates, clinician uptake, and feasibility to then inform national recommendations.
III. Radiologists often find vertebral fractures incidentally on imaging investigations (CT, MRI) taken for other purposes. To what degree are radiologists per country encouraged to identify, diagnose, and refer a patient with vertebral fractures to a FLS? This could be addressed by developing and distributing a standard addendum for radiology reports (e.g., “incidental vertebral fracture detected; FLS referral recommended”), and integrate this into the PACS/reporting systems in 2–3 hospitals per country as a quality improvement initiative.
IV. Fracture liaison services vary in availability between countries and their approaches. How to stimulate more comprehensive secondary prevention to screen and treat vertebral fractures? A potential project to address this could be mapping and benchmarking existing FLS models across participating countries using a standardized questionnaire to then publish a comparative summary with practical recommendations to support new FLS setup in countries with low coverage.
V. How will the identification of a vertebral fracture on lateral radiograph or DXA-VFA influence the choice of therapy. Is there evidence for preference for bone-anabolic treatment over anti-resorptive in patients with prevalent vertebral fracture? This could be answered by performing a retrospective chart review across centers to evaluate whether patients with identified VFs are more likely to receive anabolic treatment, and what factors influence this choice (e.g., grade, age, reimbursement).
VI. What do we know about prevalence of non-osteoporotic deformity in different parts of the world, such as short vertebral height, Scheuermann's disease, scoliosis etc.? To answer this question we could leverage existing (spine) radiographs from large cohort studies to quantify the prevalence of SVH and other non-osteoporotic deformities by age and region, and publish a visual reference atlas with examples.
VII. How to screen for otherwise unknown (asymptomatic) vertebral fractures? This could be potentially answered by embedding opportunistic VFA screening in routine DXA visits at select sites for a 6-month period and evaluate detection rates and treatment initiation.
VIII. What is the prevalence of vertebral fracture in specific high-risk groups among different ethnicities? This could be answered with an effort to form a working group to harmonize VF data from several multiethnic cohort studies using standardized adjudication, and publish cross-ethnic prevalence comparisons.

## CRediT authorship contribution statement

**Fjorda Koromani:** Writing – original draft. **Jiawei Li:** Writing – review & editing. **Hiroshi Hagino:** Writing – review & editing. **Richard Eastell:** Writing – review & editing. **Annegreet Vlug:** Writing – review & editing. **Ling Wang:** Writing – review & editing. **Hua Yue:** Writing – review & editing. **Yong-Chan Ha:** Writing – review & editing. **Steven Cummings:** Writing – review & editing. **Salvatore Minisola:** Writing – review & editing. **Claus-C. Glüer:** Writing – review & editing. **Ling Oei:** Writing – review & editing, Writing – original draft, Supervision.

## Declaration of competing interest

The authors declare that there are no conflicts of interest regarding the publication of this paper. All authors have contributed significantly to this manuscript.

## Data Availability

No data was used for the research described in the article.
